# A critical role of epigenetic inactivation of miR-9 in EVI1^high^ pediatric AML

**DOI:** 10.1186/s12943-019-0952-z

**Published:** 2019-02-27

**Authors:** Nupur Mittal, Liping Li, Yue Sheng, Chao Hu, Fuxing Li, Tongyu Zhu, Xiaohong Qiao, Zhijian Qian

**Affiliations:** 1 Department of Medicine and UI Cancer Center, University of Illinois at Chicago, Chicago, IL USA; 2 Department of Medicine and UF Health Cancer Center, University of Florida, FL32610, 2033 Mowry Road, Rm257, Gainesville, FL USA; 3 Department of Pediatrics, Division of Pediatric Hematology Oncology, Rush University Medical Center, Chicago, USA; 4 Department of Urology, Fudan University ZhongShan Hospital, Shanghai, China; 5 Department of Pediatrics, Tongji Hospital, Tongji University School of Medicine, Shanghai, China

**Keywords:** EVI1, miR9, Hyper methylation, Pediatric acute myeloid leukemia (AML), Hypomethylating agents

## Abstract

Ectopic Viral Integration site 1 (EVI1) upregulation is implicated in 10–25% of pediatric acute myeloid leukemia (AML) and has an inferior outcome with current chemotherapy regimens. Here we report that EVI1 upregulation is associated with methylation of the miR-9 promoter and correlated with downregulation of miR-9 in human AML cell lines and bone marrow (BM) cells from pediatric patients. Reactivation of miR-9 by hypomethylating agents and forced expression of miR-9 in EVI1^high^ leukemia cell lines and primary leukemia cells results in apoptosis and decreased proliferation of EVI1^high^ leukemia cells. Furthermore, re-expression of miR-9 delays disease progression in EVI1^high^ leukemia-xenograft mice. Our results suggest that EVI1-induced hypermethylation and downregulation of the miR-9 plays an important role in leukemogenesis in EVI-1^high^ pediatric AML, indicating that hypomethylating agents may be a potential therapeutic strategy for EVI1^high^ pediatric AML.

**Electronic supplementary material:**

The online version of this article (10.1186/s12943-019-0952-z) contains supplementary material, which is available to authorized users.

Ectopic Viral Integration site 1 (EVI1) was identified as a murine common locus (now designated as MECOM) of retroviral integration leading to myeloid tumors [1]. EVI1 hyperexpression has been found in up to 10–25% primary and up to 50% of secondary AML in pediatric and young adult patients, and EVI1^high^ expression in AML has been associated with poor prognosis [2, 3]. EVI1 gene encodes a zinc finger protein that functions as a transcriptional regulator in early development [1], and it is critical for hematopoietic stem cell (HSC) self-renewal as well as myeloid progenitor cell differentiation and survival in mice [4]. Multiple functional properties for EVI1 have been reported to interact with various pathways and acts as repressor of transcription [4].

MicroRNA-9 (miR-9) is deregulated in several types of solid tumors serving as an oncogene in some and tumor suppressor in others [5]. Low miR-9 expression is associated with unfavorable prognosis in adult AML [5]. MiR-9 was reported to suppress leukemic growth of t (8; 21) AML in vitro and in vivo [6]. Our previous studies illustrate a unique role for miR-9 in myelopoiesis in mouse model [7]. In the present study, we found that epigenetic regulation of miR-9 by EVI1-mediated hypermethylation plays a critical role in leukemogenesis and reversal of this process may reactivate its tumor suppressor function.

## EVI1 activation results in hypermethylation of miR-9 promoter which leads to inactivation of miR-9 in human leukemia cell lines and primary leukemia cells

**Fig. 1:**
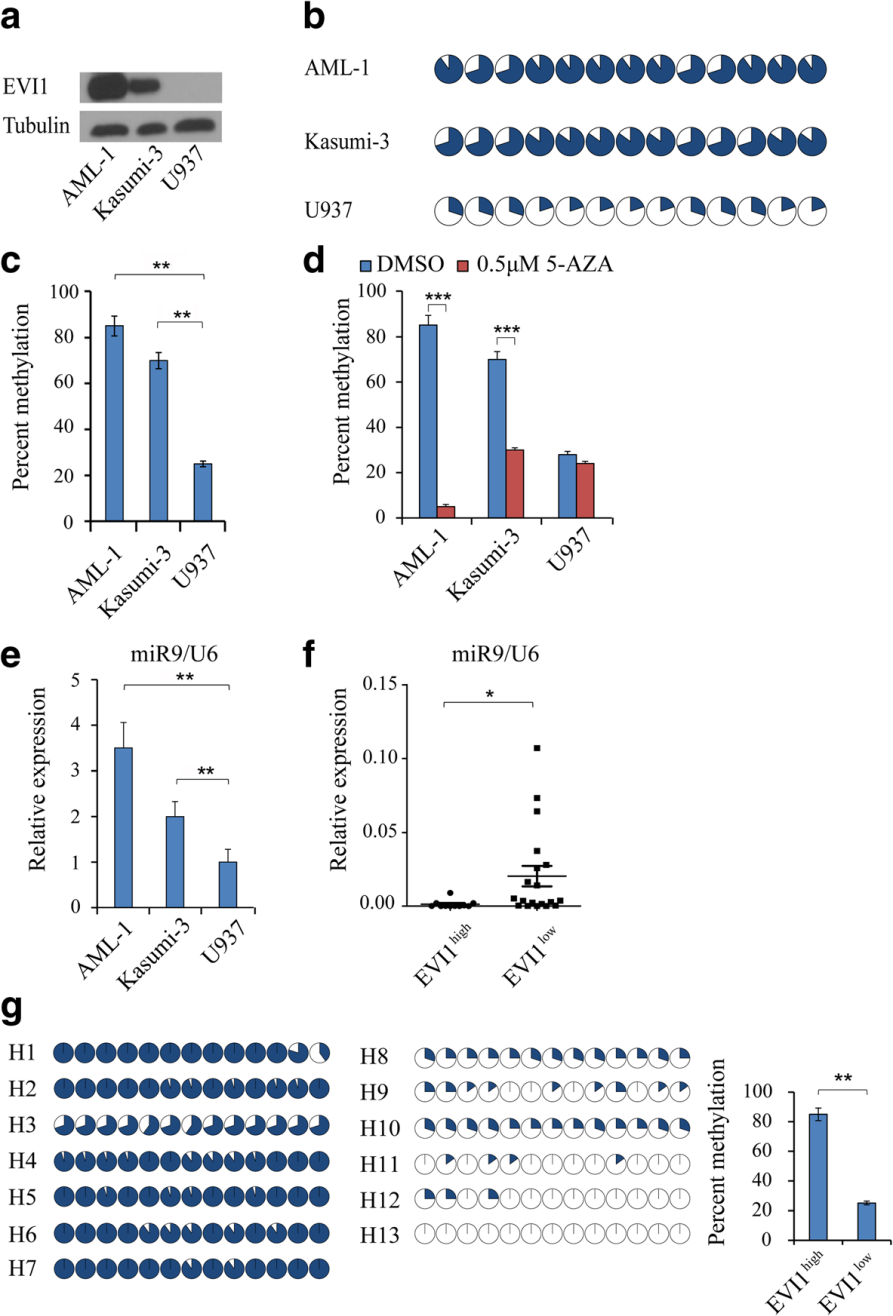
High EVI1 expression in AML cell lines and primary AML BM cells from patients is associated with hypermethylation of the miR-9 promoter. **a** EVI1 expression determined by western blot in AML cell lines. **b** DNA methylation profile of CpG dinucleotides in the promoter region of miR-9 in AML cell lines. The cycles represent the average methylation level of a specific CpG dinucleotide. Unmethylated, partially methylated, and fully methylated are indicated by open circles, partially blacked circles and blacked circles **c** Increased methylation in EVI1^high^ cell lines depicted using histogram plot. The y axis represents the percent methylation of the miR-9 promoter. **d** Histogram showing average percent methylation of the CpG islands of the miR-9 promoter in the AML cell lines before and after treatment with 5-AZA. Methylation was assessed using bisulfite conversion and direct sequencing of cell lines treated with 5-AZA for 48 h. **e** Histogram showing fold increase in miR-9 expression in EVI1^high^ cell lines AML-1 and Kasumi-3 relative to EVI1^low^ cell line, U-937 after 5-AZA treatment. **f** qRT-PCR analysis of miR-9 in pediatric primary AML BM cells. **g** Left panel: Methylation profile of promoter region of miR-9 in pediatric AML patient BM cells with EVI1^high^ (AMLH1-H7), and EVI1^low^ (AML H8-H13) expression. Right panel: Increased percent methylation in EVI1^high^ patient samples depicted using histogram. The y axis represents the percent methylation of the miR-9 promoter. All data are representative of two-to-three independent experiments. *, *P* < 0.05; **, *P* < 0.01; ***, *P* < 0.001

By Western blot analysis, we found that AML-1 and Kasumi − 3 cell lines have high EVI1 expression while U937 has low EVI1 expression (Fig. 1a). We next examined the methylation status of the miR-9 promoter region with enriched CpG islands in these cell lines. The EVI1^high^ cell lines (AML-1 and Kasumi-3) had significantly more methylation (85 and 70% respectively) versus EVI1^low^ cell line (U937) 28% (Fig. 1b and c). Next, to ascertain the effect of this EVI1 mediated hypermethylation on miR-9, we analyzed miR-9 expression by RT-qPCR in these cell lines after 48-h treatment of 5-AZA (*Decitabine*), which is a hypomethylating agent [8]. The percentage of methylation in miR-9 promoter was decreased significantly in AML-1 and Kasumi-3 but not in U937 cell line (Fig. 1d), indicating that hypermethylation of miR-9 promoter can be reversed by the hypomethylating agent. Notably, miR-9 expression was significantly increased by 5-AZA in EVI1^high^ cell lines, AML-1 and Kasumi-3 compared to the EVI1^low^ cell line U937 (Fig. 1e). Next, we examined EVI1 and miR-9 expression in primary BM cells from pediatric patients. The median EVI1 expression for the pediatric samples was noted and samples with EVI1 expression above this were regarded as EVI1^high^, whereas those with expression less than this were regarded as EVI ^low^. The details of the primary patient samples are presented in Supplementary Table 1. The detailed methods are provided in Additional file 2. Consistent with the results in cell lines, high EVI1 expression in primary patient samples correlated with low miR-9 expression (Fig. 1f). Analysis of methylation of miR-9 promoter region with enriched CpG islands in BM cells from both EVI1^high^ and EVI1^low^ patients revealed that EVI1^high^ patients had significantly more methylation of CpG islands in the miR-9 promoter compared to EVI1^low^ patients (Fig. 1g). Our findings indicate that EVI1 induces hypermethylation of miR-9 promoter, which is correlated with downregulation of miR-9 in human leukemia cell lines and primary leukemia cells.

## Reactivation of miR-9 inhibits leukemic potential of EVI1 ^high^ leukemia cell lines and primary AML cells from EVI1^high^ AML patients

**Fig. 2:**
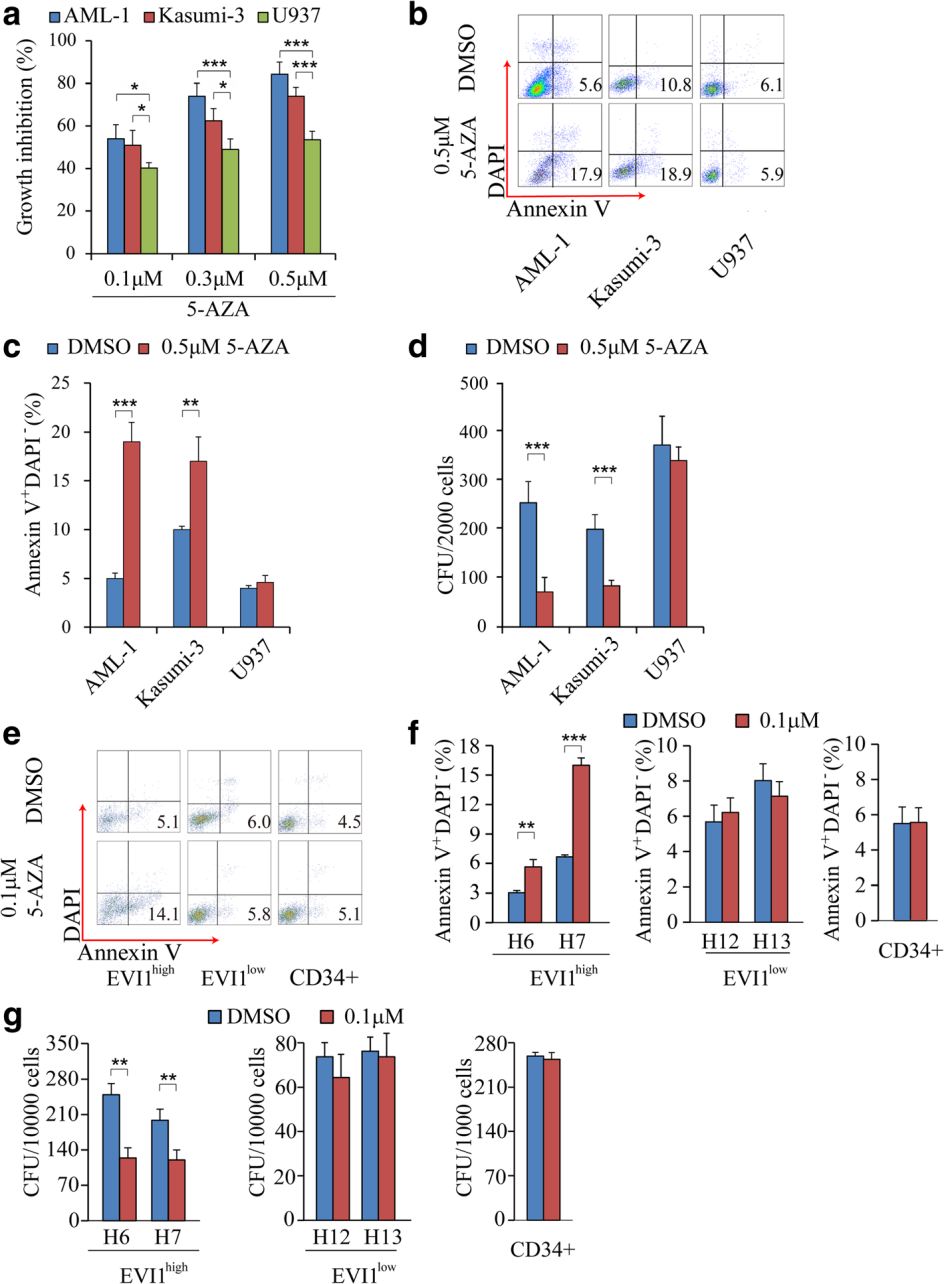
Effects of reactivation of miR-9 by 5-AZA treatment on leukemic potential of EVI1^high^ AML cell lines and primary AML cells from EVI1^high^ AML patients. **a** Analysis of growth of EVI1^high^ and EVI1^low^ cell lines after treatment with 5-AZA. Growth was assessed in liquid culture media and cell number and viability assessed using trypan blue staining. Y axis denotes percentage growth inhibition at 3 days. **b** Representative flow cytometric analysis of apoptosis in EVI1^high^ and EVI1^low^ lines treated with 5-AZA. **c** Histograms depict the frequency of apoptosis in EVI1^high^ and EVI1^low^ lines treated with 5-AZA. **d** Histograms showing the colony-forming ability of EVI1^high^ and EVI1^low^ leukemia cell lines treated with 5-AZA. **e** Representative flow cytometric analysis and **f** histogram of frequency of apoptosis after primary EVI1 ^high^ (AML sample H6 and H7) and EVI1^low^ (AML sample H12 and H13) and CD34 cells were treated with 5-AZA. **g** Histograms showing the Colony forming ability of EVI1^high^, EVI1 ^low^ primary AML cells and CD34 cells with or without treatment of 5-AZA.All data are representative of two-to-three independent experiments. *, *P* < 0.05, **, *P* < 0.01; ***, *P* < 0.001

To determine if miR-9 plays a key role in driving the oncogenic function of EVI1 in EVI1^high^ AML, we activated miR-9 expression using 5-AZA. Compared to U937 cells, the AML-1 and Kasumi-3 cells showed marked growth inhibition in response to increased concentration of 5-AZA (Fig. 2a). In addition, 5-AZA significantly induced apoptosis of AML-1 and Kasumi-3 cells but not U937 cells (Fig. 2b and c). We next examined the leukemogenic potential of these cell lines in response to 5-AZA by colony forming unit (CFU) assay. Notably, AML-1 and Kasumi-3 cells gave rise to significantly lower numbers of CFU in medium containing 5-AZA as compared to the medium without 5-AZA. In contrast, 5-AZA did not affect the cloning-forming ability of U937 (Fig. 2d, Additional file 1: Figure S1). Further examination of the effects of 5-AZA on leukemogenic potential and apoptosis of EVI1^high^ and EVI1^low^ primary patient leukemia cells as well as CD34^+^ BM cells from a healthy individual revealed that 5-AZA treatment significantly increased the apoptosis, and inhibited the colony-forming ability of EVI1^high^ patient leukemia cells but it did not affect the survival and colony-forming ability of EVI1^low^ patient leukemia cells and control CD34^+^cells (Fig. 2e, f and g).

**Fig. 3:**
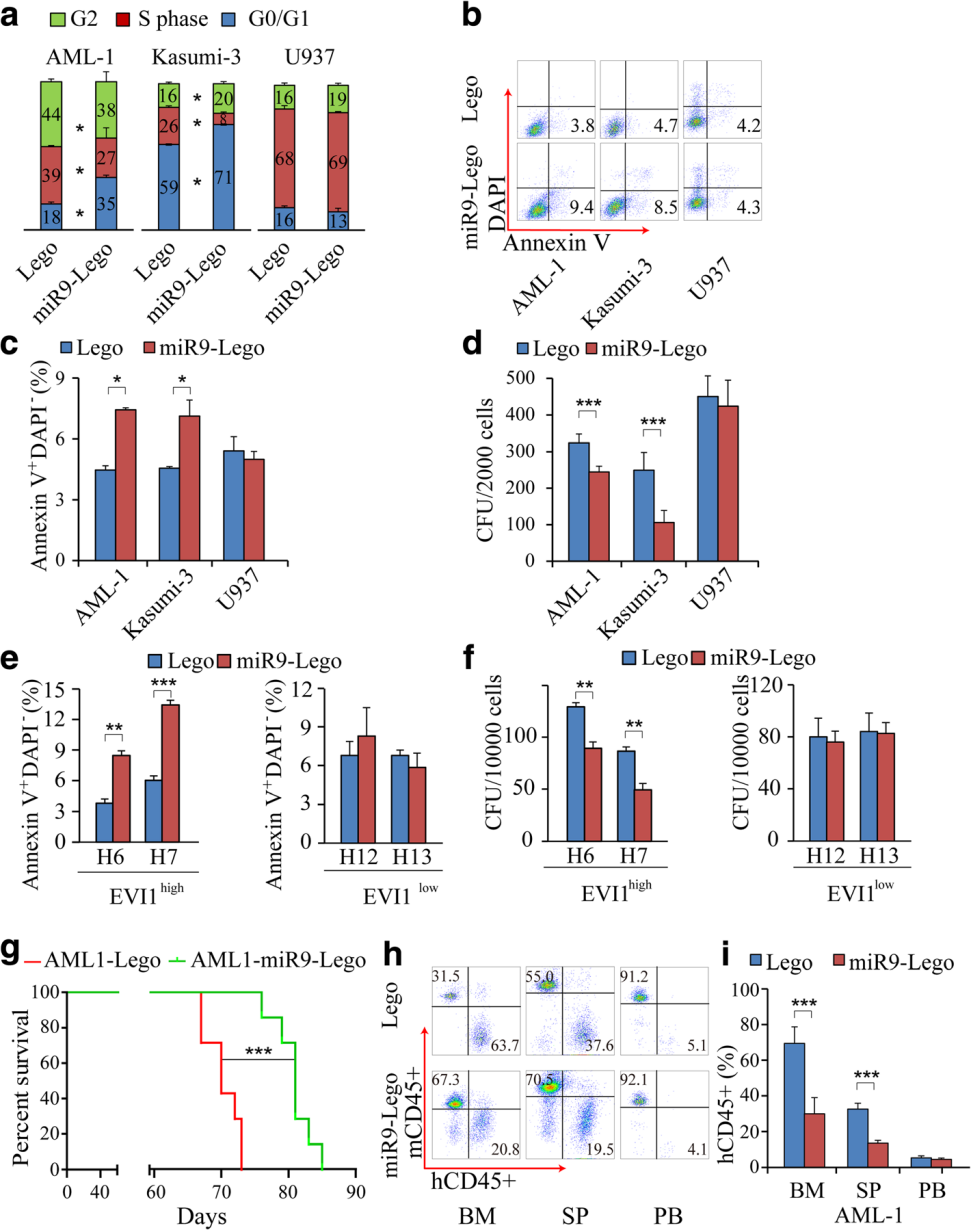
Effects of forced expression of miR-9 on leukemogenic potential of EVI1^high^ cell lines in vitro and in vivo in EVI1^high^ leukemia xenograft mice. **a** Cell cycle status of EVI1^high^ and EVI1^low^ leukemia cells with ectopic expression of miR-9 was determined by flow cytometry. Histograms depict the frequency of cells in G0/G1, S and G2 phases of cell cycle. **b** Representative flow cytometric apoptosis of EVI1^high^ and EVI1^low^ with ectopic expression of miR-9. **c** Histograms depict the frequency of apoptosis in EVI1^high^ and EVI1^low^ with ectopic expression of miR-9. **d** Colony-forming assay. AML cell lines with ectopic expression of miR-9 were cultured in methylcellulose medium and colonies and counted at 7 days of culture. **e** Frequency of apoptosis in EVI1^high^ (AML Sample H6 and H7) and EVI1^low^ (AML sample H12 and H13) cells with ectopic expression of miR-9. **f** Histograms showing the colony forming ability of EVI1^high^, EVI1^low^, primary AML cells with ectopic expression of miR-9 or vector. **g** Kaplan–Meier analysis of xenograft mice (*n* = 7) injected with AML1 cells with miR-9-Lego or Lego empty control vector. **h** Representative flow cytometric analysis of engrafted AML-1 cells (hCD45+) and mouse cells in NSG mice (*n* = 4 to 5). **i** Histogram depicts the percent AML-1 cells in bone marrow, spleen and peripheral blood after engraftment. All data are representative of two-to-three independent experiments. *, *P* < 0.05; **, *P* < 0.01; ***, *P* < 0.001

5-AZA treatment could affect the expression of many genes in the leukemia cells and hence their functions. To ascertain that the effects of 5-AZA on EVI1^high^ leukemia cells are mediated by upregulation of miR-9, we expressed miR-9 in the EVI1^high^ and EVI1^low^ cell lines using lentivirus vector. As determined by flow cytometric analysis, AML-1 and Kasumi-3 but not U937 with exogenous miR-9 expression demonstrated a significantly decreased cell population in S phase (Fig. 3a), indicating that forced expression of miR-9 inhibits proliferation of EVI1^high^ but not EVI1^low^ cells. In addition, re-expression of miR-9 induced apoptosis of AML-1 and Kasumi-3 but not U937 cells (Fig. 3b and c). AML-1 and Kasumi-3 cells with ectopic expression of miR-9 had a significantly reduced colony-forming ability as compared to those cells with expression of control vector whereas U937 cells with expression of control vector or miR-9 gave rise to comparable number of colonies (Fig. 3d, Additional file 1: Figure S2). Furthermore, ectopic expression of miR-9 enhanced the effects of 5-AZA treatment on induction of apoptosis and inhibition of growth and colony forming ability of EVI1^high^ AML1 leukemia cells (Additional file 1: Figure S3). Of note, miR-9 inhibition by a miR-9 sponge significantly rescued the effects of 5-aza on growth, apoptosis and colony-forming ability of the EVI1^high^ AML1 cells (Additional file 1: Figure S4), suggesting that miR-9 is a critical mediator of the effect of 5-aza in AML leukemia cells.

Next, we re-expressed miR-9 in these primary AML bone marrow cells from EVI1^high^ and EVI1^low^ patients using lentiviral vector. Similar to the effects of 5-AZA, re-expression of miR-9 significantly induced apoptosis (Fig. 3e) and inhibited colony-forming ability of EVI1^high^ but not EVI1^low^ primary patient leukemia cells (Fig. 3f). To determine whether ectopic expression of miR-9 has an inhibitory effect on EVI1^high^ leukemia cells in vivo, we next generated mice xenograft model with AML1 infected with miR-9-Lego or Lego empty control vector. Within 73 days post transplantation, all AML1 xenograft mice carrying control vector died (median survival time of 70 days) while all xenograft mice expressing miR-9 survived (median survival time of 81 days), indicating that ectopic expression of miR-9 delayed the disease latency in AML1 xenograft mice (Fig. 3g). When the mice became moribund, flow-cytometric analysis revealed ectopic expression of miR-9 decreased AML1 expansion in the BM, SP but not in PB (Fig. 3h and i). We generated another cohort of xenograft mice with U937 cells expressing miR-9-Lego or Lego empty control vector, and found that ectopic expression of miR-9 did not affect the growth of U937 cells in vivo as determined by flow cytometric analysis of U937 cells in BM, SP and PB in the xenograft mice (Additional file 1: Figure S5). These studies showed that similar to 5-AZA treatment, forced expression of miR-9 had an inhibitory effect on leukemogenic potential of EVI1^high^ cells, suggesting that the inhibitory effect of 5-AZA treatment on EVI1^high^ cells may be mediated by activation of miR-9 and that miR-9 plays a critical role in EVI1-induced leukemogenesis.

## Conclusion

We provide the first evidence, that miR-9 is significantly downregulated in a subset of pediatric AML patients with high expression of EVI1 and that miR-9 has a critical role in EVI1-induced leukemogenesis in pediatric patients, thereby establishing the role of miR-9 as a tumor suppressor in the pathogenesis of EVI1-induced myeloid leukemia. The molecular mechanism of miR-9 silencing in AML patients has not been elucidated. EVI1 regulates Runx1-mediated transcription activity [9] while miR-9 downregulation is associated with presence of RUNX1-ETO fusion gene in AML patients [6]. As shown in Additional file 1: Figure S6, RUNX1 knockdown in EVI1^high^ AML1 cells did not affect miR-9 expression, indicating that EVI1-induced miR-9 downregulation was not mediated by RUNX1. Our findings suggest that miR-9 downregulation is a consequence of EVI1-induced hypermethylation of miR-9 promoter in AML patients. As EVI1 was shown to interact with DNMT3, a DNA methyltransferase [10], recruitment of DNMT3 by EVI1 may participate in EVI1-induced hypermethylation of miR-9. These findings could inform the direction for future clinical trials to evaluate the role of hypomethylating agents in therapy for EVI1^high^ pediatric and young adult AML.

## Additional files

Additional file 1: Supplementary table and figures. (ZIP 244 kb)

Additional file 2: Supplementary methods. (DOCX 19 kb)

## Abbreviations

5-AZA: Decitabine
AML: Acute myeloid leukemia
BM: Bone marrow
CFU: Colony forming units
EVI1: Ectopic Viral Integration site 1
miR-9: MicroRNA-9
PB: Peripheral blood
RT-qPCR: Real time quantitative Polymerase chain reaction
SP: Spleen
