# Supplementary material for: A critical role of epigenetic inactivation of miR-9 in EVI1high pediatric AML
Source: Mol Cancer. 2019 Feb 27;18:30. doi: 10.1186/s12943-019-0952-z (PMC6391809; doi:10.1186/s12943-019-0952-z)
Supplement: Supplementary file 1 — Supplementary table and figures. (ZIP 244 kb) [file 12943_2019_952_MOESM1_ESM.zip › 12943_2019_952_MOESM1_ESM/Supplementary Figures.pdf]

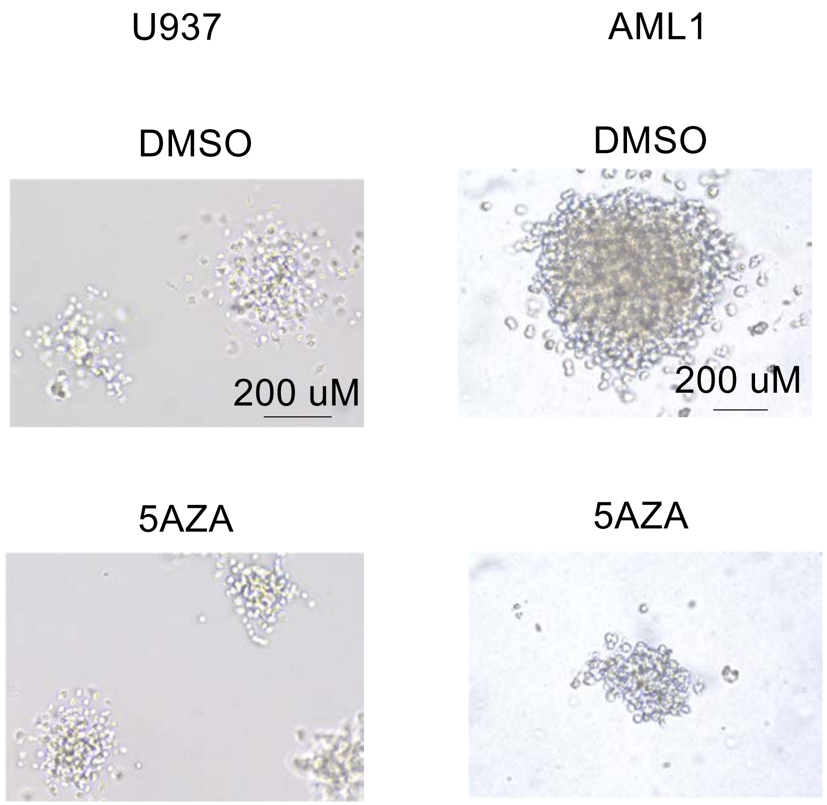

Figure S1. Representative colonies for AML1 and U937 cells with 5'-AZA treatment. U937 or AML1 human leukemia cells were plated in methylcellulose medium containing DMSO or 0.5 μM 5-AZA. 7-10 days after plating, the colonies were analyzed.

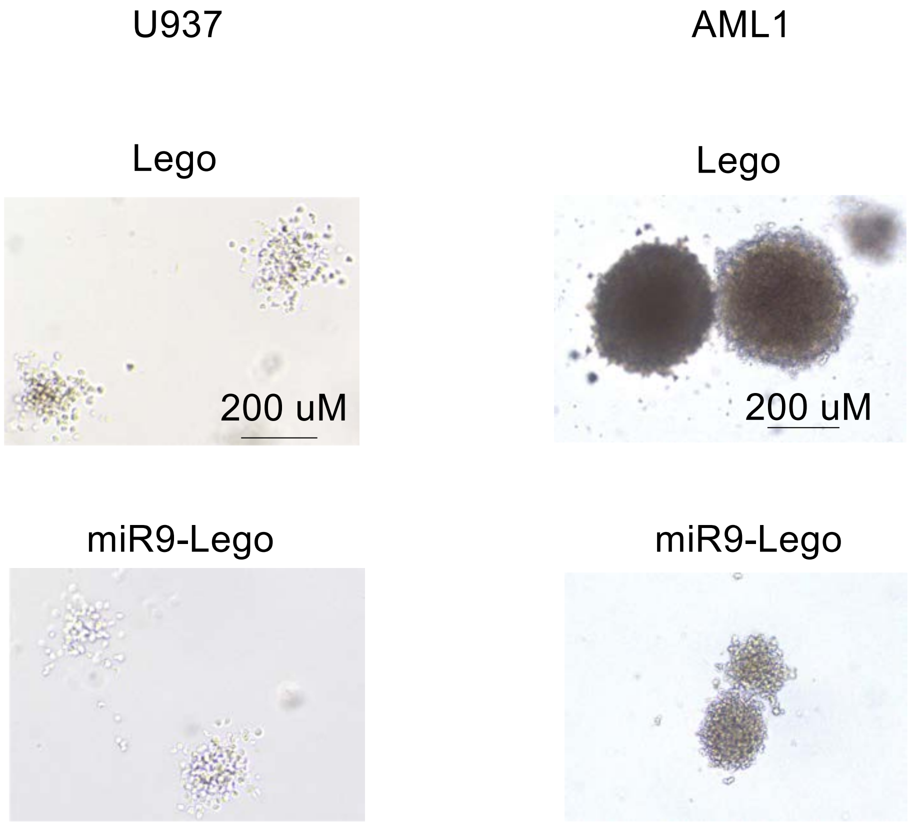

Figure S2. Representative colonies for AML1 and U937 cells with forced expression of miR-9. U937 or AML1 human leukemia cells with expression of miR-9 or control vector Lego were plated in methylcellulose medium. 7-10 days after plating, the colonies were analyzed.

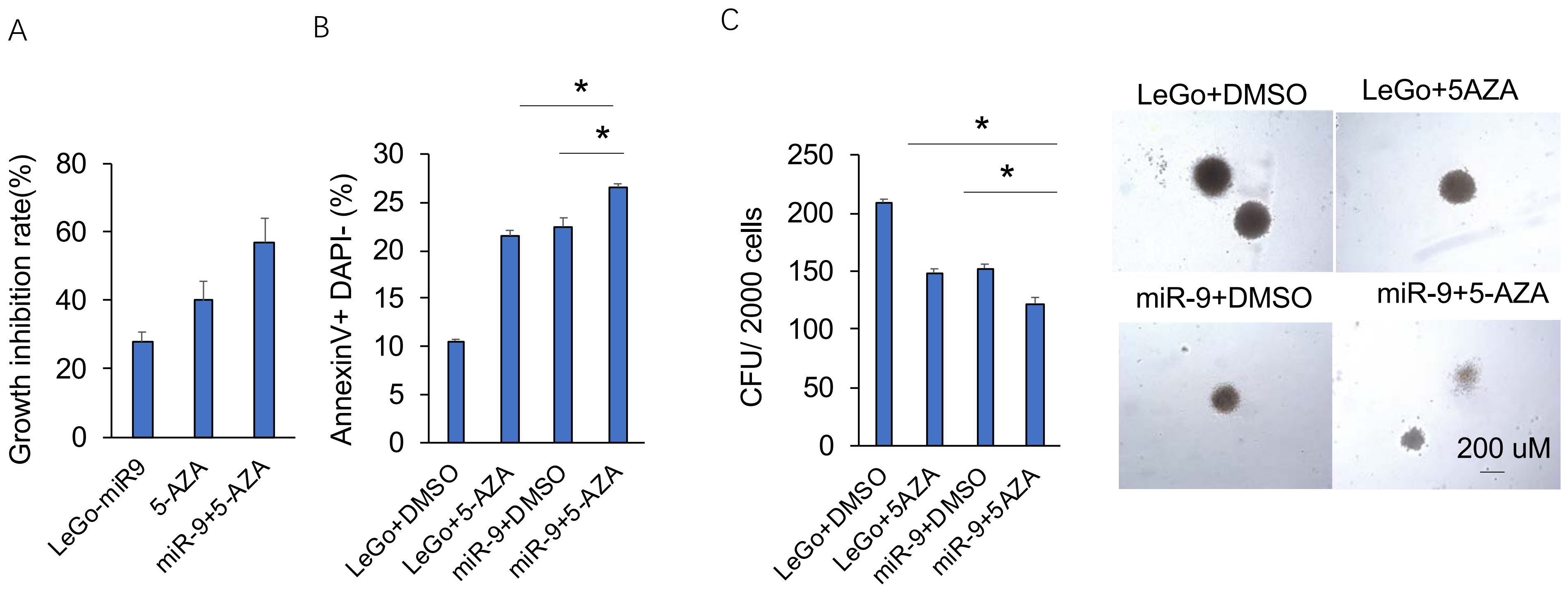

Figure S3. Combination of enforced expression of miR-9 and 5'-AZA treatment has an addictive effects on growth inhibition, apoptosis and colony-forming ability of AML1 cells. Cell growth (A), Apoptosis (B) of AML1 cells in liquid culture and Colony forming Unit (C) of AML1 cells in methylcellulose medium were determined.

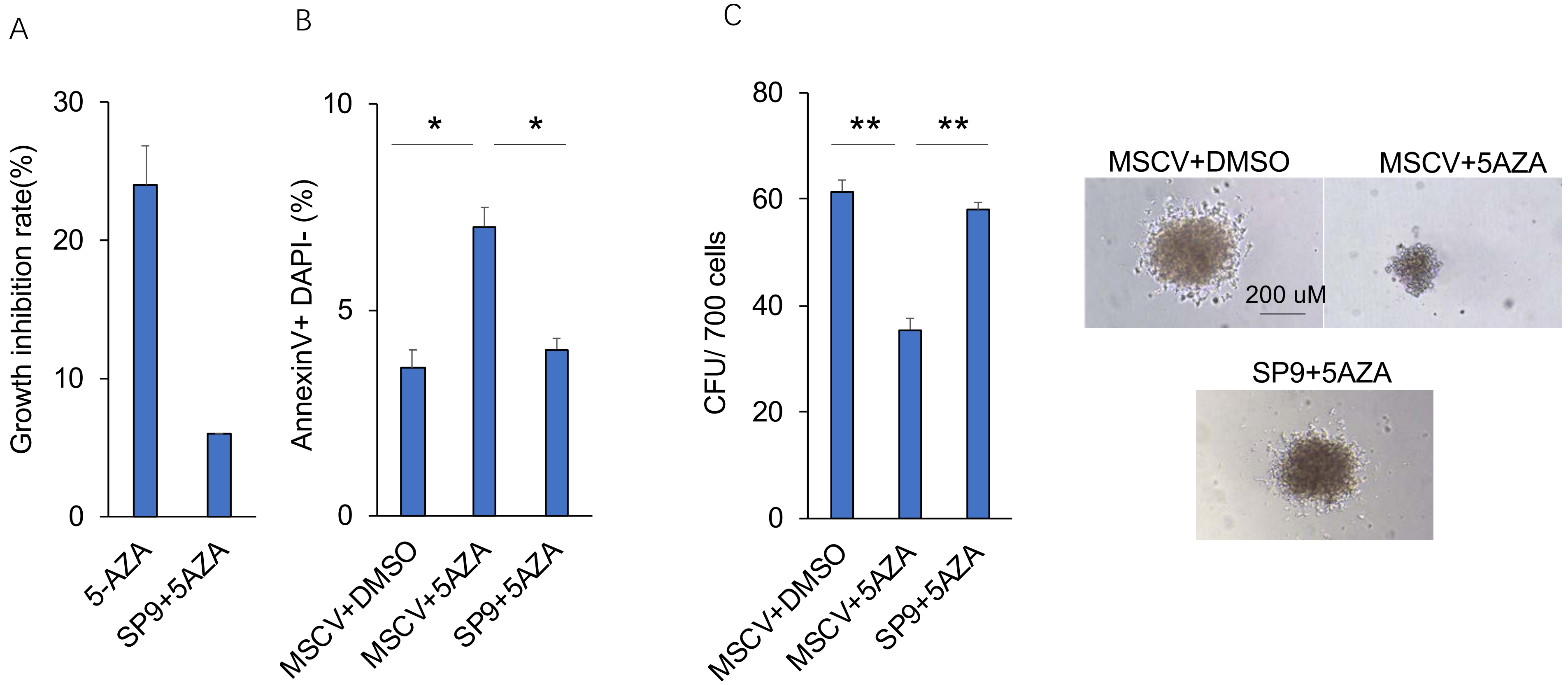

Figure S4. Inhibition of miR-9 rescues the effects of 5-AZA treatment on growth inhibition, apoptosis and colony forming ability of AML1 leukemia cells. The miR-9 sponge (SP9) was expressed in MSCV retroviral vector. AML1 leukemia cells were infected with retrovirus expressing MSCV vector or SP9. Cell growth (A), Apoptosis (B) of AML1 cells in liquid culture and Colony forming Unit (C) of AML1 cells in methylcellulose medium with or without 5'-AZA treatment were determined..

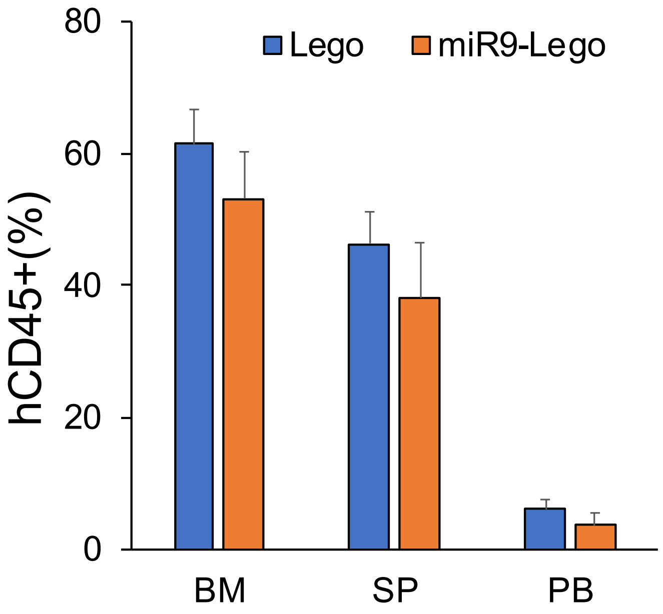

Figure S5. Histogram depicts the percent U-937 cells in bone marrow (BM), spleen (SP) and peripheral blood (PB) after engraftment. The U937 cells expressing miR-9 or Lego control vector were transplanted into NSGS mice. The percentage of U937 cells was determined by flow cytometric analysis in different tissues from the xenograft mice 2-3 weeks after transplantation (N=3-5 mice).

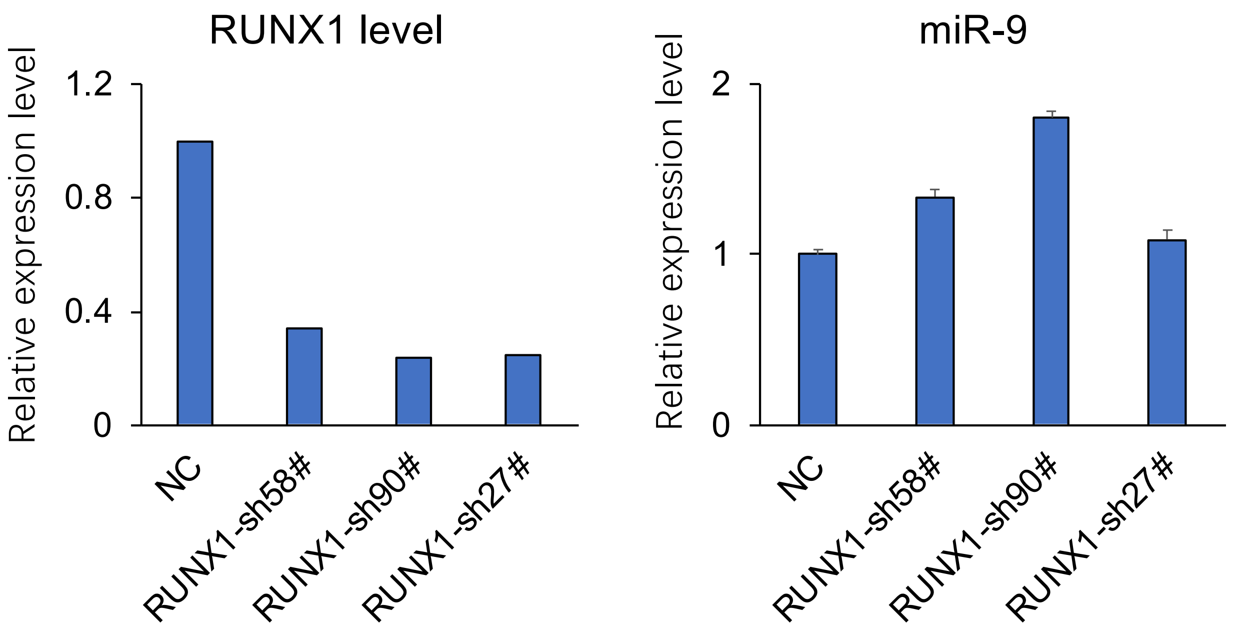

Figure S6: Knockdown RUNX1 does not affect expression level of miR-9 in AML1 cells. The AML1 cells were infected with lentiviral vector expressing shRNAs against RUNX1. The expression of miR-9 and RUNX1 expression was determined by real-time qPCR.
