# Supplementary material for: A critical role of epigenetic inactivation of miR-9 in EVI1high pediatric AML
Source: Mol Cancer. 2019 Feb 27;18:30. doi: 10.1186/s12943-019-0952-z (PMC6391809; doi:10.1186/s12943-019-0952-z)
Supplement: Supplementary file 2 — Supplementary methods. (DOCX 19 kb) [file 12943_2019_952_MOESM2_ESM.docx]

**Supplementary methods**

**Cell lines and patient samples**

AML-1 cell line was purchased from DSMZ (German collection of microorganisms and cell cultures) bank while Kasumi-3 and U-937 were purchased from ATCC in 2014. These cell lines were expanded and cryogenically frozen upon acquisition to establish stocks that were stored in liquid nitrogen until use. The cell lines were cultured within 3-6 months after resuscitation. Human cord blood cells were obtained from New York Blood center and CD34+ cells were isolated. All patients provided written informed consent in accordance with the Declaration of Helsinki. Institutional Review Board approval was obtained at the University Of Illinois Medical Center. AML-1, Kasumi-3 and U937 cell lines were grown according to the company instructions. The human CD34 cells were maintained in Stem Span culture medium (Sigma) supplemented with human SCF 10 ng/ml, Human TPO 20 ng/ml, FGF-1 10ng/ml, ANGPTL 3 (angiopoietin like family of growth factors) 500ng/ml (Peprotech, USA) and heparin 10ng/ml.

### In vitro culturing of primary AML cells

Cryopreserved AML samples were rapidly thawed at 37°C and washed with phosphate-buffered saline containing 0.1% human serum albumin. The Human AML bone marrow (BM) mononuclear cells (MNCs) were cultured in IMDM supplemented with 30% FBS and 1% P/S and L-glutamine. Growth factors were kept at concentrations of 20 ng/ml recombinant human stem cell factor, 10 ng/ml recombinant human GM- CSF, 10 ng/ml recombinant human IL-3 and 20 ng/ml human TPO. All cytokines were purchased from Peprotech. In experiments using cryopreserved leukemic cells, the cells were grown for 24 h before transduction.

**RNA isolation, cDNA synthesis, and real-time PCR**

Total RNA was extracted from the MNC fraction of BM or peripheral blood from normal donors and leukemia patients and of cell lines using Trizol (Invitrogen), and cDNAs were obtained with Taqman microRNA reverse transcript kit (Applied Biosystems). Total RNAs isolated from AML patients and cell lines were transcribed into cDNA using Superscript III reverse transcriptase (Invitrogen), and were subjected to the qRT-PCR. Primers used are EVI1**-** F: 5’- TTGCCAAGTAACAGCTTTGCTG -3’, R: 5’- CCAAAGGGTCCGAATGTGACTT -3’ . Taqman primers for miR-9 and U6 were purchased from Thermo Scientific.

**Plasmids**

Lego-IG was purchased from Addgene and murine miR-9 (90bp) was cloned from genomic DNA and inserted in EcoRI and Not1 site of Lego-IG vector. PLKO.1-RUNX1 shRNA plasmids were purchased from Sigma. SP9, a sponge miR-9 was previously described[1].

**Mice**

6-8 weeks NSGS mice (NOD.Cg-Prkdcscid Il2rgtm1Wjl Tg(CMV-IL3, CSF2, KITLG) 1Eav/MloySzJ) were obtained from Jackson lab. AML1 cells were injected by i.v. NSGS mice after 2.5 Gray irradiation. Mice were monitored every day. Peripheral blood samples were collected from the tail vein. All animals are free to access food and water. All animal research was approved by the University of Illinois at Chicago and University of Florida Institutional Animal Care and Use Committee.

**Flow cytometric analysis**

Suspended single cells were collected from bone marrow, spleen and peripheral blood. Cells were incubated with antibodies in FACS buffer (2%FBS in PBS) on ice for 20 minutes at dark. Human CD45 and mouse CD45 antibodies were purchased from eBioscience. All cells were analyzed by Flow cytometry on CyAn bench-top analyzer (Beckman Coulter).

**Western Blot**

Anti-EVI1 primary antibodies (Cell signaling technology) and anti-tubulin primary antibodies (Millipore Corporation) were used followed with peroxidase-conjugated secondary antibodies (Cell Signaling Technology, USA).

**Apoptosis and BrdU Cell cycle analysis**

For the detection of apoptosis, the stably transfected AML cell lines were stained with annexin V antibody (e- Biosciences) and 4′,6-diamidino-2-phenylindole (DAPI; 1:1000). For cell cycle analysis, all the cells were labeled with 30ug/ml BrdU overnight, followed by fixation and permeabilization with Cytofix/Cytoperm (BD Biosciences), treatment with DNase I (Sigma), and staining with a BrdU-specific antibody (Bu20a; Ebioscience). The cells were analyzed by flow cytometry with a CyAn ADP flow cytometer. Data analysis was performed with FlowJo Analysis Software.

**Primers used in bisulfite sequencing**

For bisulfite sequencing, the forward primer (5’-GTTTGTTTATTTTTTTTGGTTTTT -3’) and reverse primer (5’- AACCTCCCTTAACCAATACC-3’) were used for PCR to amplify the regions containing the CpG islands. MiR-9 CpG Island was amplified from bisulfite treated genomic DNA by PCR.

**Statistics**

In general, results are expressed as the mean ± standard deviation (SD) for triplicate experiments. The Student t test was used to compare differences between groups. *P* values of < 0.05 were regarded as statistically significant. For survival data, Log-rank (Mantel-Cox) test was used for the analysis. For Figs. 1c, 1e and 2a, one-way Annova followed by post hoc Dunnett’s multiple comparison test was used for analysis.

1. Senyuk V, Zhang Y, Liu Y, Ming M, Premanand K, Zhou L, Chen P, Chen J, Rowley JD, Nucifora G, Qian Z: **Critical role of miR-9 in myelopoiesis and EVI1-induced leukemogenesis.** *Proc Natl Acad Sci U S A* 2013, **110:**5594-5599.
